# Account of Diseases in an Eastern District of London

**Published:** 1802-04

**Authors:** 


### ACCOUNT of DISEASES in an EASTERN DISTRICT of LONDON,

*From February* 20, *to March* 20, 1802.

| | |
|---|---|
| **ACUTE DISEASES.** | |
| Typhus Mitior | 2 |
| Pneumonia | 4 |
| Peripneumonia Notha | 10 |
| Dyſenteria | 5 |
| Rheumatiſmus Acutus | 3 |
| **CHRONIC DISEASES.** | |
| Catarrh | 4 |
| Tuſſis | 33 |
| Dyſpnœa | 10 |
| Tuſſis cum Dyſpnœa | 12 |
| Hydrothorax | 4 |
| Pleurodyne | 5 |
| Palpitatio | 2 |
| Apoplexia | 1 |
| Paralyſis | 2 |
| Vertigo | 3 |
| Cephalalgia | 6 |
| Vomitus | 2 |
| Dyſpepſia | 7 |
| Enterodynia | 2 |
| Colic | 1 |
| Conſtipatio | 3 |
| Dyſuria | 4 |
| Hæmorrhois | 2 |
| Herpes | 3 |
| Lumbago | 3 |
| Rheumaticus Chronicus | 21 |
| **PUERPERAL DISEASES.** | |
| Ephemera | 2 |
| Menorrhagia Lochialis | 4 |
| Maſtodynia | 3 |
| Prolapſus Vaginæ | 1 |
| **INFANTILE DISEASES.** | |
| Rubeola | 5 |
| Pertuſſis | 6 |
| Aphthæ | 3 |
| Ophthalmia | 2 |

The obſervations reſpecting the rapid changes of the weather, which were made in the laſt Report, may with propriety be repeated in the preſent. During the laſt few weeks, the wind has blown from very different points, and the temperature of the air has been much varied. The ſame ſpecies of diſeaſe, therefore, as might well be expected, ſtill prevail. The attention of medical practitioners has of late been very much engaged by the diſeaſes of children: A great number of this claſs of patients have been viſited by the meaſles, the ſymptoms of which have, in ſome inſtances, appeared in their moſt threatening form. In the mildeſt form of this diſeaſe, the fever generally abates after the eruption has appeared; but in the caſes referred to, the fever continued, and was accompanied with ſuch ſymptoms of pneumonic inflammation as to excite conſiderable alarm, and, in ſome inſtances, proved fatal. When we remember that a mild diſeaſe ſometimes ſucceeds a conſiderable degree of what has been called the eruptive fever, our prognoſis reſpecting the termination of the diſeaſe cannot with any propriety be formed previouſly to the eruption, and muſt then be determined, principally, by the degree to which the thoracic affection prevails. It has been a queſtion whether blood-letting, which is a powerful means of reducing inflammation, ſhould be had recourſe to in the early part of the diſeaſe or towards wards the close of it. This should be determined rather by the degree of inflammation than by that of eruptive fever, since, as it has been observed, a considerable degree of fever often precedes a mild disease, in which the cough and other pneumonic symptoms do not wear a threatening aspect. The bloodletting therefore has in some cases, with great propriety, been deferred to a later period of the disease, even till after the desquamation has taken place, at which time the fever and cough, and other symptoms of thoracic inflammation, often appear in their most alarming form, and soon put a fatal period to life; or else lay a foundation for a more permanent affection of the lungs, terminating in the equally fatal phthisis pulmonalis.

